# Reference Values and Determinants of Fractional Exhaled Nitric Oxide in a Representative Adult Population in Western Sweden

**DOI:** 10.1002/clt2.70107

**Published:** 2025-09-18

**Authors:** Reshed Abohalaka, Selin Ercan, Lauri Lehtimäki, Saliha Selin Özuygur Ermis, Daniil Lisik, Muwada Bashir Awad Bashir, Radhika Jadhav, Linda Ekerljung, Göran Wennergren, Jan Lötvall, Teet Pullerits, Helena Backman, Madeleine Rådinger, Bright I. Nwaru, Hannu Kankaanranta

**Affiliations:** ^1^ Krefting Research Centre Department of Internal Medicine and Clinical Nutrition Institute of Medicine Sahlgrenska Academy University of Gothenburg Gothenburg Sweden; ^2^ Allergy Centre Tampere University Hospital Tampere Finland; ^3^ Faculty of Medicine and Health Technology Tampere University Tampere Finland; ^4^ Department of Internal Medicine/Respiratory Medicine and Allergology The Sahlgrenska Academy University of Gothenburg Gothenburg Sweden; ^5^ Department of Paediatrics Sahlgrenska Academy University of Gothenburg Gothenburg Sweden; ^6^ Department of Public Health and Clinical Medicine Umeå University Umeå Sweden; ^7^ Department of Respiratory Medicine Seinäjoki Central Hospital Seinäjoki Finland

**Keywords:** FE_NO_, fractional exhaled nitric oxide, normal range, population‐representative, reference values

## Abstract

**Background:**

Fractional exhaled nitric oxide (FE_NO_) is used to differentiate asthma inflammatory phenotypes and guide its management. However, data on FE_NO_ reference values in a representative adult population is limited. We aim to derive reference values and determinants of FE_NO_ in a representative adult population.

**Methods:**

The West Sweden Asthma Study is a clinical‐epidemiological population‐representative study of randomly selected adults in Western Sweden. From this cohort, 943 subjects participated in comprehensive clinical investigations, including skin prick testing (SPT), specific immunoglobulin E (sIgE) analysis, and FE_NO_ measurement. Clinical allergy was defined as co‐occurrence of atopy (positivity to SPT or sIgE) and self‐reported allergic symptoms to the same allergen family. FE_NO_ levels were analysed in relation to the presence or absence of clinical allergy, asthma, and other factors.

**Results:**

The 95^th^ percentile of FE_NO_ ranged from 34 parts per billion (ppb) in those between 30 and 40 years old to 52 ppb in those ≤ 30 years old in the entire sample (*N* = 943), and from 26 to 37 ppb in those without clinical allergy, asthma, or chronic obstructive pulmonary disease (COPD) (*n* = 587), depending on age. Sex, smoking, clinical allergy, atopy, asthma, and hypertension influenced FE_NO_ levels, meanwhile, age, asthma, clinical allergy, and reversibility‐related variables were significant determinants of FE_NO_ levels.

**Conclusion:**

The 95^th^ percentile (upper normal limit) for FE_NO_ ranges from 34 to 52 ppb overall, and from 26 to 37 ppb in those without clinical allergy, asthma, or COPD, depending on age. These findings provide a guide for interpreting FE_NO_ in the general population.

**Abbreviations**
BMI: body mass index
COPD: chronic obstructive pulmonary disease
FE_NO_
: fractional exhaled nitric oxide
FEV_1_
: forced expiratory volume in one second
FVC: forced vital capacity
GINA: Global Initiative for Asthma
GOLD: Global Strategy for the Diagnosis, Management and Prevention of Chronic Obstructive Lung Disease
ICS: inhaled corticosteroids
ppb: parts per billion
sIgE: specific immunoglobulin E
SPT: skin prick test
WSAS: West Sweden Asthma Study

## 1 Introduction

Nitric oxide is a cellular signalling molecule that plays a pivotal role in the physiological regulation of respiratory tract function and inflammatory processes [1, 2]. Measurement of fractional exhaled nitric oxide (FE_NO_) correlates with induced‐sputum eosinophilia [3] as well as with airway hyperresponsiveness [3, 4]. Furthermore, the measurements are characterized by ease of execution and high patient acceptance [5]. FE_NO_ thus stands as a promising biomarker differentiating asthma from other respiratory diseases [6, 7]. While using FE_NO_ to confirm an asthma diagnosis remains a subject of controversy [2, 8], it is acknowledged as a valuable biomarker for identifying asthma phenotypes and guiding asthma treatment [9, 10]. Elevated FE_NO_ levels are associated with poor asthma control [4, 11], as well as reduction in FE_NO_ levels following inhaled steroid treatment [12, 13]. Beyond asthma, elevated FE_NO_ levels are seen in chronic obstructive pulmonary disease (COPD) patients [14] and a correlation between FE_NO_ levels and sputum eosinophils in smoking COPD patients has been reported [15]. These observations highlight the potential of FE_NO_ as a marker for Type 2 (T2) airway inflammation [3, 16, 17].

Many of the few reports that have attempted to establish reference values for FE_NO_ in the general population are not population representative studies [18, 19]. Meanwhile, the rest confine their allergy definition exclusively to atopy [20, 21], omit upper limits of normal, exclude smokers, or neglect to examine the effects of allergy at all [18, 22, 23, 24], thus ignoring clinical variables that may exert a significant influence on FE_NO_ levels. These studies were able to detect an overlap in FE_NO_ values between their ‘healthy’ individuals and those suffering from asthma. Consequently, guidelines recommended the adoption of cut‐off values instead of reference values for interpreting FE_NO_ levels. For instance, the American Thoracic Society (ATS) suggests using cut‐offs of FE_NO_ > 25 and > 50 parts per billion (ppb) to determine the response to corticosteroid therapy in adults as indeterminate or likely, respectively [25]. According to the Global Initiative for Asthma (GINA), a FE_NO_ concentration ≥ 20 ppb as part of systematic assessment of difficult‐to‐treat asthma under regular medication is considered high, along with other biomarkers indicative of T2 immune response [26]. The European Respiratory Society (ERS) recommends a cut‐off of 40 ppb for diagnostics [27]. The determination of these cut‐off points does not derive from reference values established within the general population [28]. Instead, the selection of these cut‐off values is predicated upon achieving an optimal balance in relation to the correlation between FE_NO_ levels and sputum eosinophils, as well as in the context of diagnosing asthma within studies with relatively modest sample sizes [28, 29]. Furthermore, various factors influence FE_NO_ values in healthy populations, including age, height, ethnicity, sex, atopy and smoking [18, 20, 21, 30, 31]. Despite this, prevailing clinical guidelines advocate for either age‐specific threshold values [28] or a singular cut‐off point for interpreting FE_NO_ results [26].

Therefore, this work aimed to ascertain the reference values of FE_NO_ based on a representative adult population in Western Sweden, while considering the impact of various allergic and non‐allergic, inflammatory and non‐inflammatory factors.

## 2 Methods

### 2.1 Study Area and Population

The West Sweden Asthma Study (WSAS) is a population‐based cohort study, which has been previously described in detail [32, 33]. Briefly, WSAS follows randomly selected individuals aged 16–75 years at baseline. Initiated in 2008, 30,000 subjects within this age group were chosen from the Swedish Population Register to participate in a postal survey. The selection process considered age and gender stratification to ensure a representative sample of the population in Västra Götaland county (comprising nearly a fifth of the total Swedish population).

**FIGURE 1:**
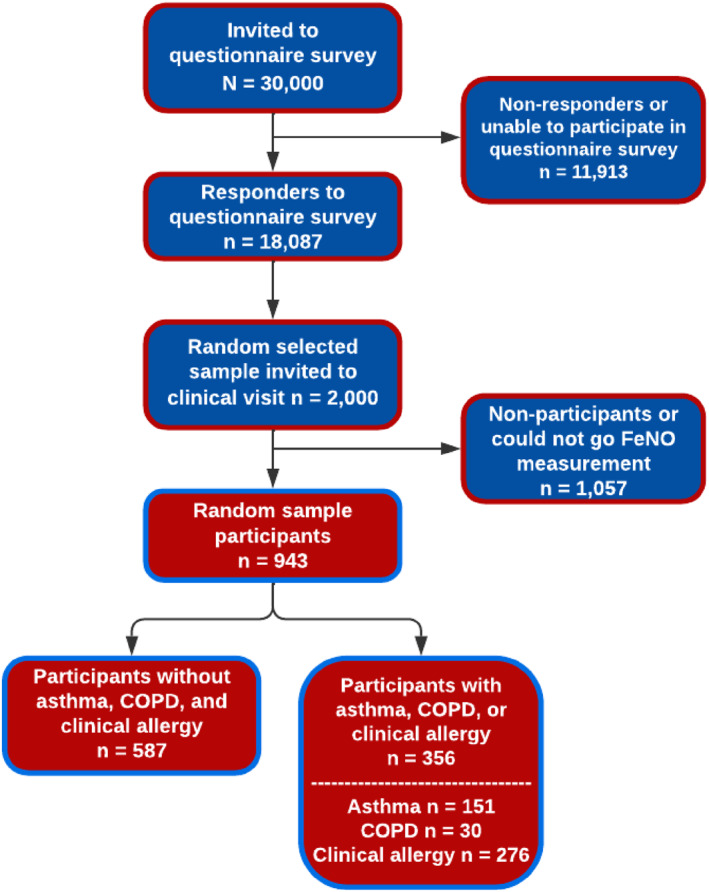
The flowchart illustrating the establishment of participant cohorts emanates from a systematically sampled populace in the Western Sweden Asthma Study (WSAS).

From the survey responders, a random subset of 2000 subjects was invited to undergo comprehensive clinical examinations, and 1172 took part between 2009 and 2012. Ultimately, 943 participants successfully completed all clinical measurements, including the measurement of FE_NO_ (Figure 1). Non‐responders did not differ in prevalence estimates of airway diseases or symptoms when compared with responders [34]. All participants signed an informed consent to the study protocol, approved by the regional ethics board in Gothenburg, Sweden.

### 2.2 Clinical Examinations

The clinical investigations included haematological examinations for blood differential cell counts, allergic sensitization evaluations through skin‐prick tests (SPTs) and specific immunoglobulin E (sIgE) levels, anthropometric assessments involving measurements of height and weight, determination of FE_NO_, spirometry, and methacholine challenge. In addition, clinical interviews were conducted, along with the administration of questionnaires including, but not limited to, respiratory diseases, allergy, symptoms, and comorbidities.

### 2.3 Measurement of FE_NO_

FE_NO_ was assessed using an electrochemical device (NiOX VERO, Aerocrine, Morrisville, NC, USA). These FE_NO_ measurements were conducted prior to spirometry. The analyser sensor was replaced after 300 measurements or one year, whichever came first. All procedures adhered to the latest ATS/ERS guidelines [35, 36]. During the measurement, subjects exhaled against a mouth pressure of 5 cm H_2_O aiming at a flow rate of 50 mL/s. NO concentration was measured during the last 5 s of a 10 s period when flow rate was between 45 and 55 mL/s. Participants were asked to avoid eating and using tobacco products for 2 h before the measurements. If the FE_NO_ measurement did not meet the predefined quality criteria [35, 36], the measurement was repeated until an acceptable result was obtained.

### 2.4 Assessment of Sensitization and Clinical Allergy

Sensitization was assessed through the determination of sIgE levels and/or skin prick tests for 11 aeroallergens (Details are provided in the Supporting Information S1). *Clinical allergy* was defined by the existence of sensitization and self‐reported allergic symptoms when exposed to the matching sensitized allergen family [37]. These symptoms, evaluated during the clinical interview prior to the sensitization test results, included ocular manifestations, nasal discomfort, various forms of allergic nasal expressions, pruritus in the oral or pharyngeal region, respiratory challenges, exacerbation of asthma symptoms, pruritic skin rash, and disruptions in gastrointestinal function.

### 2.5 Definitions of Asthma

*Asthma* was defined as meeting any of the following criteria: (1) documented diagnosis of asthma by a healthcare professional or a history of asthma accompanied by respiratory symptoms or use of asthma medication in the past 12 months, (2) respiratory symptoms or use of asthma medication in the past 12 months, coupled with a positive reversibility test (12% and 200 mL increase in FEV_1_), or (3) respiratory symptoms or use of asthma medication in the past 12 months, accompanied by a positive methacholine challenge indicative of asthma (i.e., participants with moderate to high hyperreactivity were considered to have asthma even if not previously diagnosed). Healthy individuals were those free from asthma, COPD, and clinical allergies.

### 2.6 Statistical Analyses

When setting reference values, we assume that the range of results from a group of healthy people represents what is normal. People whose results fall outside this normal range—usually defined as the middle 90% (between the 5th and 95th percentiles)—are often considered to have unusual or abnormal results [38]. As a result, our reporting includes the various percentiles of FE_NO_ values, with a specific emphasis on the upper limit of normal, defined as the 95^th^ percentile. Because FE_NO_ values showed a right‐skewed distribution, we applied a logarithmic transformation to the dataset, following the method described previously [39]. Percentiles were then calculated from the transformed data for the entire sample and for each 10‐year age group. Afterwards, the percentile values were converted back to their original scale and used to plot the percentile curves.

Multiple linear regression analyses were conducted using enter method to discern factors associated with higher FE_NO_ values considering potential confounders. Mean comparisons were depicted with corresponding standard deviations and assessed employing independent Student's t‐tests. Statistical significance was determined at *p* < 0.05. All statistical analyses were performed using SPSS 29.0 (IBM Corp, New York, USA).

## 3 Results

### 3.1 Characteristics of the Random Sample

The random sample, which underwent comprehensive clinical investigation, comprised 943 individuals (mean age = 50.5 years, SD = 15.5), with 47.7% (*n* = 450) being males. More than half of the participants (51.6%, *n* = 487) reported no history of smoking, while 10.8% (*n* = 102) were current smokers. For further insights into lung function, FE_NO_, allergic variables, and comorbidities among participants with or without clinical allergy, see Table 1.

**TABLE 1:** Characteristics of WSAS I random sample subjects with or without asthma and clinical allergy (*N* = 927)^b^.

	Without clinical allergy		With clinical allergy	
Subjects	With asthma	Without asthma	With asthma	Without asthma
*n* (%)	50 (7.7)	601 (92.3)	101 (36.6)	175 (63.4)
Demographics				
Age (y)	55 (16)	52 (15)	45 (14)	45 (15)
Male sex *n* (%)	20 (40.0)	271 (45.1)	46 (45.5)	105 (60.0)
BMI, kg/m^2^	26.6 (3.7)	26.0 (4.1)	26.8 (4.8)	26.1 (4.1)
Education years over 12 *n* (%)	23 (46.0)	266 (44.3)	59 (59.0)	93 (53.4)
Never smokers	22 (44.0)	290 (48.3)	53 (52.5)	114 (65.1)
Current smokers	6 (12.0)	71 (11.8)	11 (10.9)	12 (6.9)
Pack‐year mean (SD)^a^	19.3 (17.0)	14.2 (12.7)	15.7 (16.1)	12.4 (14.0)
FENO (ppb)				
Median (IQR)	16.0 (18.9)	15.8 (10.9)	19.9 (19.0)	19.3 (13.8)
Mean (SD)	25.4 (26.6)	17.9 (10.1)	27.8 (24.8)	23.4 (15.0)
Range (min‐max)	4.6–127.7	0.1–102.9	6.4–176.0	5.1–98.8
Lung function				
Pre‐bronchodilator FEV_1_ (% of predicted)	87.7 (19.3)	101.3 (13.6)	90.4 (16.1)	99.1 (12.2)
Pre‐bronchodilator FVC (% of predicted)	95.0 (14.5)	102.5 (13.2)	97.2 (12.4)	101.4 (11.4)
Pre‐bronchodilator FEV_1_/FVC	0.73 (0.11)	0.79 (0.06)	0.75 (0.08)	0.79 (0.07)
Post‐bronchodilator FEV_1_ (% of predicted)^c^	96.8 (21.9)	103.9 (14.3)	93.7 (17.7)	103.4 (12.5)
Post‐bronchodilator FVC (% of predicted)^c^	101.9 (15.3)	102.0 (13.9)	98.1 (12.8)	102.1 (11.7)
Post‐bronchodilator FEV_1_/FVC^c^	0.74 (0.11)	0.80 (0.07)	0.76 (0.09)	0.81 (0.07)
Post‐bronchodilator FEV_1_—pre‐bronchodilator FEV_1_ (L)^c^	0.21 (0.14)	0.11 (0.11)	0.24 (0.18)	0.13 (0.12)
Reversibility %^c^	10.3 (9.6)	3.9 (4.0)	9.1 (9.7)	3.9 (3.7)
Atopy and allergy				
Positivity to skin prick test (SPT)^d^	4 (13.8)	43 (11.3)	86 (98.9)	137 (91.9)
Positivity to phadiatop‐IgE	4 (8.0)	49 (8.5)	93 (95.9)	153 (91.6)
Atopic (SPT or sIgE)	6 (12.0)	69 (11.5)	101 (100)	175 (100)
Clinical allergy to mite	—	—	38 (37.6)	35 (20.0)
Clinical allergy to furry animals	—	—	63 (62.4)	54 (30.9)
Clinical allergy to pollen	—	—	82 (81.2)	135 (77.1)
Clinical allergy to nuts	—	—	41 (42.3)	31 (18.6)
Clinical allergy to seafood	—	—	17 (17.5)	31 (18.6)
Morbidities				
COPD	5 (10.0)	14 (2.3)	7 (6.9)	2 (1.1)
Obesity (BMI ≥ 30 kg/m^2^)	8 (16.0)	89 (14.8)	25 (24.8)	26 (14.9)
Diabetes mellitus	3 (6.0)	17 (2.8)	6 (5.9)	10 (5.7)
Hypertension	18 (36.0)	144 (24.1)	23 (23.1)	31 (17.8)
Hyperlipidaemia	10 (20.0)	94 (15.6)	13 (12.9)	26 (14.9)
Metabolic disease	23 (46.0)	239 (39.8)	44 (43.6)	62 (35.4)

*Note:* Data was presented as *n* (%) or mean (SD).

Abbreviations: BMI = body mass index, FEV_1_ = Forced expiratory volume in 1 s, FEV_1_% = Percentage of predicted normal value, FVC = Forced vital capacity, Smoking history, pack‐y = pack years of smokers.

^a^ Evaluated for those reporting being ever‐smokers.

^b^ Some participants (*n* = 16) did not undergo skin prick testing or IgE measurement. As a result, we could not determine their clinical allergy status, and they were excluded from Table 1.

^c^ Some participants underwent methacholine challenge and were excluded from the post bronchodilation measurement, the valid number of participants for the post bronchodilation are (*n* = 30, 338, 54, and 86) respectively for each group.

^d^ Some participants could not undergo SPT therefore the valid number of participants are (*n* = 29, 382, 87, and 149) respectively for each group.

### 3.2 Fractional Exhaled Nitric Oxide in the Random Sample

**FIGURE 2:**
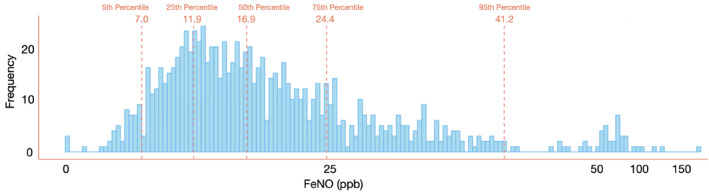
Frequency distribution of Fractional exhaled nitric oxide (FE_NO_) in the entire random sample. The dotted lines indicate the 5^th^, 25^th^, 50^th^, 75^th^ and 95^th^ percentiles.

The distribution of FE_NO_ levels within the random sample was right‐skewed, as depicted in Figure 2. Consequently, percentiles were calculated from the dataset after logarithmic transformation, with a median value of 16.9 ppb. The lower and upper normal limits corresponding to the 5^th^ and 95^th^ percentiles were determined to be 7.0 and 41.2 ppb, respectively.

**FIGURE 3:**
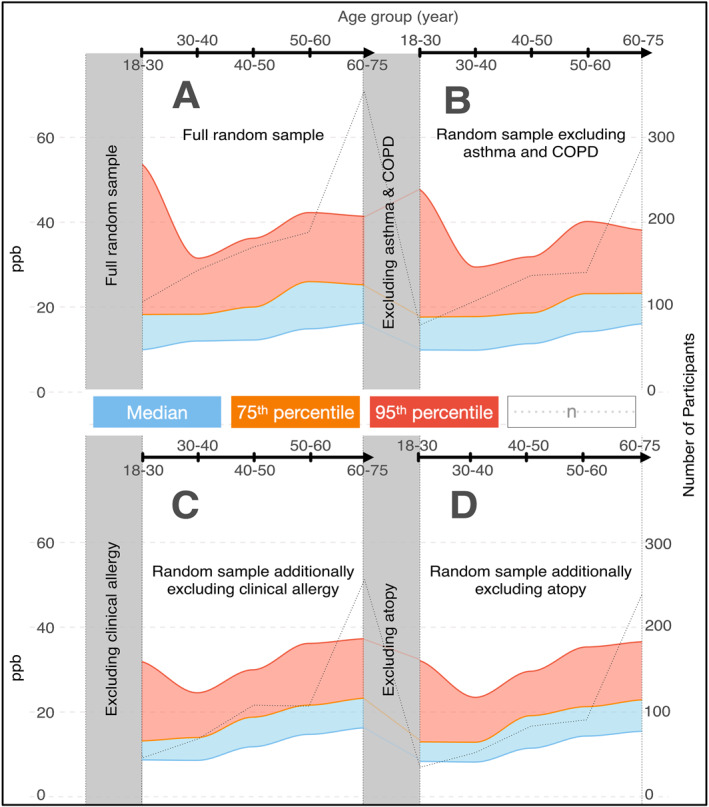
The characteristics of FE_NO_ across different age groups, delineated for (A) the entire random sample (*N* = 943), (B) random sample participants excluding asthma and COPD patients (*n* = 773), (C) individuals additionally devoid of clinical allergy (*n* = 587), and (D) individuals additionally devoid of atopy (*n* = 519).

The upper normal limit of FE_NO_ within the entire sample exhibited a gradual increase with increasing age after 30 years old, meanwhile, it decreased from 52.2 ppb for 18–30 years age group to 34.0 ppb for 30–40 years age group (Figure 3A). To ascertain the influence of asthma and COPD on FE_NO_ levels, individuals diagnosed with either condition were excluded from the analysis (Figure 3B). Remarkably, upper normal limit of FE_NO_ showed quite similar pattern as in the whole population. Following this, we aimed to explore the impact of clinical allergy on FE_NO_ levels by additionally excluding these with clinical allergy (Figure 3C). The upper normal limit reduced among individuals without clinical allergy, asthma, and COPD, particularly within the 18–30 years age group, and was highest (37.0) among the 60+ years age group. Lastly, participants lacking atopy had a similar FE_NO_ profile as those without clinical allergy (Figure 3D).

The 75^th^ percentile and the median of FE_NO_ showed similar patterns of change to the 95^th^ percentile with age and for these without asthma, COPD, or clinical allergy in our cohort. However, unlike the 75^th^ percentile and the median, the upper normal limit decreased more remarkably in individuals without clinical allergy, asthma, and COPD, especially those aged 18 to 30. Therefore, we selected individuals without clinical allergy, asthma, and COPD as the “healthy” cohort for our study. For FE_NO_ reference values in different subgroups of our cohort, see Supporting Information S1: Figure S1.

### 3.3 Factors Affecting Fractional Exhaled Nitric Oxide in the Random Sample

A comprehensive understanding of the determinants affecting the upper normal limit of FE_NO_ within the general population necessitates a comparative investigation of FE_NO_ upper 95^th^ percentile values across diverse demographic and clinical variables. However, the sample size in our study was insufficient to draw statistically significant conclusions from such comparisons. However, when comparing the upper 95^th^ percentile values of clinical variables with adequate sample sizes, the mean showed a similar pattern (Supporting Information S1: Figure S1B). Moreover, the upper 95^th^ percentile is a collective outcome and not individually available for each patient. Hence, we opted to conduct a comparative analysis of FE_NO_ mean values to ascertain the directional impact of various demographic and clinical variables previously identified to influence FE_NO_ levels. Our investigation revealed notable disparities in FE_NO_ levels across different subgroups. Specifically, FE_NO_ levels were significantly higher in males than in females. This difference persisted even when comparing participants of both sexes within the same age and height categories. FE_NO_ levels were significantly lower among both ever and current smokers than among never and non‐current smokers. Additionally, FE_NO_ levels were significantly higher among individuals diagnosed with clinical allergy and those with atopy than among those without such conditions, as well as among individuals with asthma than individuals without asthma. Furthermore, our results showed that FE_NO_ levels were elevated in these with hypertension than those without (Table 2).

**TABLE 2:** Mean FE_NO_ levels of participants in WSAS random sample according to demographical and clinical features (*n* = 943).

Condition	With the condition	Without the condition	*p* value
Demographics			
Male sex	24.0 (16.3)	17.2 (12.6)	**<** **0.001**
Ever smoking	19.1 (11.5)	21.7 (17.4)	**0.003**
Current smoking	13.5 (8.3)	21.3 (15.3)	**<** **0.001**
Atopy and allergy			
Positivity to skin prick test	23.8 (19.4)	16.8 (9.3)	**<** **0.001**
Positivity to phadiatop‐IgE	24.7 (19.0)	18.4 (12.3)	**<** **0.001**
Atopy	23.8 (18.3)	18.4 (12.2)	**<** **0.001**
Clinically allergic to mite	26.1 (23.5)	19.9 (14.0)	**<** **0.001**
Clinically allergic to furry animals	26.4 (20.3)	19.6 (13.9)	**<** **0.001**
Clinically allergic to pollen	25.9 (20.4)	18.8 (12.5)	**<** **0.001**
Clinically allergic to nuts	25.6 (19.1)	20.1 (14.7)	**<** **0.001**
Clinically allergic to seafood or dairy	29.4 (20.2)	20.0 (14.7)	**<** **0.001**
Having at least one clinical allergy	25.0 (19.3)	18.5 (12.1)	**<** **0.001**
Comorbidities			
Obesity (BMI ≥ 30 kg/m^2^)	19.7 (10.6)	20.6 (15.6)	0.255
Asthma	27.0 (25.2)	19.2 (11.5)	**< 0.001**
COPD	17.7 (13.0)	20.5 (15.0)	0.153
Diabetes mellitus	18.0 (7.8)	20.6 (15.2)	0.152
Hypertension	22.2 (15.3)	19.9 (14.8)	**0.021**
Hyperlipidaemia	21.7 (16.5)	20.2 (14.6)	0.124
Metabolic disease	21.3 (15.7)	19.9 (14.3)	0.074

*Note:* Data was presented as mean (SD). Statistical significances were evaluated by independent samples *t*‐test. Bold font indicates *p*‐value < 0.05 which was considered statistically significant.

Abbreviations: BMI = body mass index, COPD = chronic obstructive pulmonary disease, IgE = immunoglobulin E.

### 3.4 Multiple Linear Regression Model for Determinants of FE_NO_ Levels

Multiple linear regression analyses were conducted to ascertain the factors associated with increases in FE_NO_ values within the general population, while considering potential covariates based on previous knowledge. The findings indicated that age, asthma, and clinical allergy—although not atopy—emerged as significant determinants of FE_NO_ levels across the entire sample (Table 3). Additionally, lung function parameters, such as the percentage of post‐FEV_1_ of predicted, the disparity between post‐ and pre‐FEV_1_ in litres, and the percentage of reversibility post bronchodilation test, were identified as significant determinants of FE_NO_ levels across the entire sample.

**TABLE 3:** Determinants of FE_NO_ levels in multiple linear regression analysis.

	Coefficient B	Std. Error	Lower 95% CI	Upper 95% CI	*p* value
Demographics					
Age	0.937	0.371	0.207	1.666	**0.012**
Male sex	−0.029	3.337	−6.584	6.526	0.993
Height	0.229	0.431	−0.618	1.076	0.595
Weight	−0.261	0.384	−1.016	0.494	0.497
BMI	0.622	1.139	−1.616	2.860	0.585
Ever smoking	−1.736	1.320	−4.329	0.858	0.189
Current smoking	−3.231	2.069	−7.295	0.834	0.119
Comorbidities					
Asthma	4.857	1.714	1.490	8.225	**0.005**
COPD	−3.877	4.859	−13.420	5.667	0.425
Hypertension	0.162	1.722	−3.220	3.544	0.925
Diabetes mellitus	−6.529	3.611	−13.622	0.565	0.071
Atopy	0.088	3.712	−7.204	7.381	0.981
Clinical allergy	5.607	2.448	0.798	10.416	**0.022**
Biomarkers					
Skin prick test positivity	−1.139	3.237	−7.498	5.220	0.725
Phadiatop‐IgE positivity	2.786	2.547	−2.216	7.788	0.274
Lung function^a^					
Pre‐bronchodilator FEV_1_ pred %	1.976	1.384	−0.743	4.695	0.154
Pre‐bronchodilator FVC	25.717	22.169	−17.827	69.262	0.247
Pre‐bronchodilator FVC pred %	0.558	1.446	−2.282	3.398	0.700
Pre‐bronchodilator FEV_1_/FVC	207.493	149.752	−86.659	501.644	0.166
Post‐bronchodilator FEV_1_	10.473	11.040	−11.212	32.158	0.343
Post‐bronchodilator FEV_1_ pred %	−3.232	1.597	−6.370	−0.094	**0.044**
Post‐bronchodilator FVC	−28.803	22.808	−73.604	15.998	0.207
Post‐bronchodilator FVC pred %	0.585	1.518	−2.397	3.567	0.700
Post‐bronchodilator FEV_1_/FVC	−58.906	158.921	−371.067	253.255	0.711
Post‐bronchodilator FEV_1_/FVC pred %	65.375	170.610	−269.747	400.497	0.702
Post‐bronchodilator ‐ pre‐bronchodilator FEV_1_	60.837	27.086	7.633	114.041	**0.025**
Reversibility %	1.856	0.736	0.410	3.303	**0.012**
*R*: 0.489, *R* Square: 0.240, adjusted *R* Square: 0.203					

*Note:* Variables linked with the elevation of FE_NO_ were examined through linear regression analyses encompassing the entire randomized sample (*n* = 943). Data are presented as the unstandardized *b* coefficient, standard error of *b*, and 95% CI. Bold font indicates *p*‐value < 0.05 which was considered statistically significant.

Abbreviations: FEV_1_, Forced expiratory volume in 1 s; FEV_1_%, Percentage of predicted normal value; FVC, Forced vital capacity.

^a^ Pre‐bronchodilator FEV_1_ and pre‐bronchodilator FEV_1_/FVC predicted variables were excluded from the regression analysis due to multicollinearity reasons.

**FIGURE 4:**
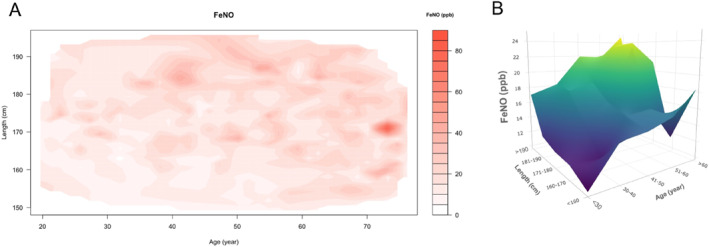
The distribution of FE_NO_ among participants categorized by age and height, excluding those participants with asthma, COPD, and clinical allergy. The distribution is presented in two formats: (A) continuous variables of age and height represented through a heatmap displaying FE_NO_ levels, and (B) age and height grouped variables through a surface expressing the mean value of FE_NO_.

In addition, we explored the determinants of FE_NO_ levels excluding individuals with asthma, COPD, and allergies. Our analysis using multiple linear regression demonstrated that, in the absence of asthma, allergies or COPD, nearly all variables listed in Table 3 were significant determinants of FE_NO_ levels (Supporting Information S1: Table S1). Within this subgroup, FE_NO_ levels were found to be influenced by both age and height, increasing with the elevation of either variable (Figure 4).

## 4 Discussion

In our study, the 95^th^ percentile of FE_NO_ ranged from 34 to 52 ppb in the entire sample and from 26 to 37 ppb among individuals without clinical allergy, asthma, and COPD, depending on age. Sex, smoking, clinical allergy, atopy, asthma, and hypertension influenced FE_NO_ levels in the total random sample. Additionally, age, asthma, clinical allergy, the percentage of predicted post‐bronchodilator FEV_1_, and reversibility in the bronchodilatation test were significant determinants of FE_NO_ levels across the full sample. These results provide reference values and patterns of FE_NO_ levels in the general population considering various background factors and relevant conditions/comorbidities. This is crucial since FE_NO_ is a key potential biomarker for T2‐airway inflammation in asthma and COPD patients [7, 15, 16, 17].

Few studies have attempted to establish reference values for FE_NO_ among adults. The upper limit of FE_NO_ in non‐smoking individuals without allergies ranged from 37.4 to 47.4 ppb [21] and from 20 to 45 ppb [22] depending on gender and from 38.3 to 62 ppb depending on height and gender [40]. In more stringent criteria, where participants were free from infections and medication usage as well, the upper limit was 19.7 ppb [41]. However, there are disparities in participant numbers and the scope of statistical variables considered across these studies. Furthermore, the sample selection process in these studies is not optimal, rendering them non‐representative of the general population and limiting their value [28].

Olin et al. [24] conducted a study aimed at formulating an equation to ascertain the upper limit of normal FE_NO_ levels within a cohort devoid of atopic individuals, thus representing a “healthy” population. Their investigation revealed a notable variability in the anticipated upper limit of normal FE_NO_ levels, ranging from 24 to 54 ppb, contingent upon the age and height of the participants. Toren et al. [42] found that normal FE_NO_ levels in adults vary between 30.3 and 50.6 ppb, depending on age and gender. Among individuals with a negative Phadiatop test, the upper limit was 36.7 ppb. Conversely, Brody et al. [18] also documented that within the adult population, the upper limit of normal FE_NO_ levels, identified using the 97.5^th^ percentile, spanned from 39.5 to 47.7 ppb, with variations correlated with age. These ranges slightly differ from the reference value documented in our study. This discrepancy could potentially arise from methodological differences. Notably, Olin et al. and Brody et al. excluded individuals with a history of smoking, a factor known to reduce FE_NO_ levels, which could potentially skew the variability in FE_NO_ levels, rendering it unrepresentative of the general population. The inclusion of smokers in the determination of FE_NO_ reference values is essential, since smoking is associated with the T2‐low asthma phenotype [43]. Moreover, Brody et al. employed exclusion criteria that solely encompassed participants with respiratory conditions and symptoms, while allergic or atopic individuals, who may exhibit elevated FE_NO_ levels, were not excluded. Conversely, our study aimed to establish the upper limit of normal FE_NO_ levels in a healthy population defined by more practical criteria as the absence of asthma, COPD, and clinical allergy, resulting in a range of 26–37 ppb, depending on age.

Although age, height and sex are widely reported as influential factors on FE_NO_ levels [18, 21, 44], Olin et al. [24] found no discernible differences between males and females of similar age and height. Conversely, Travers et al. [21] and Taylor et al. [44] consistently reported elevated FE_NO_ levels in males. Our study corroborates these findings, revealing significantly higher FE_NO_ levels among males within the entire random sample, even when comparing participants of both sexes within identical age and height categories. Moreover, the significance remained after adjusting FE_NO_ levels between males and females for height using a generalized linear model. This underscores the potentially clinically significant impact of sex on FE_NO_ levels.

One of the strengths of our study lies in its comprehensive approach to data collection, which involved a wide array of clinical assessments and measurements. By conducting thorough clinical examinations and employing standardized protocols in assessing asthma and allergy parameters, we ensured the reliability and accuracy of our data. Furthermore, our sample size was large, based on a representative cohort of individuals from the general population, thereby enhancing the generalizability of our findings. Moreover, our study addressed the limitations of previous research by considering a broader range of demographic and clinical variables, including smoking status, allergic sensitization, and respiratory conditions such as asthma and COPD. By examining the influence of these factors on FE_NO_ levels, we provided valuable new insights into the determinants of FE_NO_ variability within the general population. Despite its strengths, our study has several limitations. First, the cross‐sectional data of this study limits our ability to establish causality between the identified factors and FE_NO_ levels. Prospective studies would be beneficial in elucidating the temporal relationships between these variables. Second, we could not exclude lower airway type 2 inflammation in participants. To our knowledge, no FE_NO_ reference value study has done this, as it may require a biopsy or sputum sample analysis. Lastly, we reported FE_NO_ values using only the NIOX VERO device. Measurements with other devices may vary. However, our findings remain valid for cohorts using NIOX VERO. Notably, this device showed no FE_NO_ values exceeding the upper normal limit when compared to others in the recent Global Lung Function Initiative reference values [45].

Different guidelines suggest using cut‐off values rather than reference values for interpreting FE_NO_ levels. These cut‐offs vary, from 20 to 50 ppb depending on the clinical question, to indicate high FE_NO_ levels and a possible T2 immune response [26, 27, 28]. These cut‐off points are not based on reference values from the general population [28]. Our findings indicate that some of these clinically used cut‐offs fall within the reference values for the general population. This suggests that these cut‐offs are more about classification or treatment response rather than being purely abnormal. Additionally, our results demonstrate that some of these cut‐offs may align with reference values from the general population, suggesting the utility of them in judging abnormality. Furthermore, COPD patients with T2 inflammation have higher FE_NO_ levels due to the high production of NO by inducible NOS. However, non‐T2 COPD patients generally have lower FE_NO_ levels due to increased activity of arginase that deprives NO of its major biochemical substrate (L‐arginine) [46]. Establishing reference values specifically for COPD patients could be valuable for COPD clinics. However, our study design does not allow this, as the number of COPD patients in our cohort was too small.

In conclusion, the 95^th^ percentile of FE_NO_ ranges from 34 to 52 ppb overall, and from 26 to 37 ppb in those without clinical allergy, asthma, or COPD, depending on age. Factors such as sex, smoking, clinical allergy, atopy, asthma, and hypertension affect FE_NO_ levels. Age, asthma, clinical allergy, and reversibility are significant predictors of FE_NO_ in the general population. These findings are useful for interpreting FE_NO_ measurements, considering various factors and conditions. They also provide a guide for interpreting FE_NO_ in asthma clinics.

## Author Contributions

**Reshed Abohalaka:** conceptualization, investigation, writing – original draft, methodology, validation, visualization, writing – review and editing, software, formal analysis, data curation. **Selin Ercan:** investigation, writing – review and editing, software, data curation, formal analysis. **Lauri Lehtimäki:** investigation, conceptualization, data curation, formal analysis, validation, writing – review and editing. **Saliha Selin Özuygur Ermis:** writing – review and editing, formal analysis, software, data curation. **Daniil Lisik:** writing – review and editing, formal analysis, software, data curation. **Muwada Bashir Awad Bashir:** visualization, software, formal analysis, data curation. **Radhika Jadhav:** writing – review and editing, data curation. **Linda Ekerljung:** writing – review and editing, methodology, validation, formal analysis, resources, data curation, software, project administration, investigation. **Göran Wennergren:** writing – review and editing, formal analysis, validation, data curation, investigation. **Jan Lötvall:** writing – review and editing, software, formal analysis, data curation, resources, project administration, investigation. **Teet Pullerits:** writing – review and editing, formal analysis, data curation, investigation. **Helena Backman:** investigation, formal analysis, data curation, supervision, writing – review and editing, methodology. **Madeleine Rådinger:** investigation, writing – review and editing, methodology, validation, resources, supervision, project administration. **Bright I. Nwaru:** conceptualization, investigation, funding acquisition, writing – review and editing, methodology, validation, formal analysis, data curation, supervision, resources, project administration. **Hannu Kankaanranta:** project administration, data curation, supervision, resources, formal analysis, validation, writing – review and editing, methodology, funding acquisition, conceptualization, investigation, software.

## Conflicts of Interest

R.A. reports travel grants from the European Respiratory Society (ERS), American Thoracic Society (ATS), the Swedish heart and lung foundation (HLF), and the Adlerbertska foundation. L.L. reports personal fees from ALK, AstraZeneca, Berlin Chemie, Boehringer‐Ingelheim, Chiesi, GSK, Novartis, Orion Pharma and Sanofi outside the current work. S.S.Ö.E. reports conference‐attendance related costs from Thermo Fisher Scientific outside the current work. T.P. reports fees for lectures and/or consulting from AstraZeneca, Chiesi, GSK, Novartis and Sanofi outside the current work. H.B. reports personal fees for lectures form AstraZeneca, Boehringer Ingelheim and GSK outside the current work. B.I.N. reports personal fees for lectures and consulting from DBV Technologies and AstraZeneca outside the current work. H.K. reports fees for lectures and/or consulting from AstraZeneca, Boehringer‐Ingelheim, Chiesi, Covis Pharma, GSK, MedScape, MSD, Novartis, Orion Pharma and Sanofi outside the current work. The rest of the authors have no conflict of interest to declare.

## Supporting information


Supporting Information S1

## Data Availability

The authors have nothing to report.
