# Supplementary material for: Reference Values and Determinants of Fractional Exhaled Nitric Oxide in a Representative Adult Population in Western Sweden
Source: Clin Transl Allergy. 2025 Sep 18;15(9):e70107. doi: 10.1002/clt2.70107 (PMC12445423; doi:10.1002/clt2.70107)
Supplement: Supplementary file 1 — Supporting Information S1 [file CLT2-15-e70107-s001.docx]

**Reference values and determinants of fractional exhaled nitric oxide in a population-representative adult sample in Western Sweden**

Reshed Abohalaka^a^ MSc, Selin Ercan^a^ MD, Lauri Lehtimäki^b,c^ MD PhD, Saliha Selin Özuygur Ermis^a^ MD, Daniil Lisik^a^ MD, Muwada Bashir Awad Bashir^a^ MD PhD, Radhika Jadhav^a^ MD, Linda Ekerljung^a,d^ MD PhD, Göran Wennergren^e^ MD PhD, Jan Lötvall^a^ MD PhD, Teet Pullerits^a^ MD PhD, Helena Backman^f^ PhD, Madeleine Rådinger^a^ PhD, Bright I. Nwaru^a^ PhD, Hannu Kankaanranta^a,c,g^ MD PhD

^a^Krefting Research Centre, Department of Internal Medicine and Clinical Nutrition, Institute of Medicine, Sahlgrenska Academy, University of Gothenburg, Gothenburg, Sweden

^b^Allergy Centre, Tampere University Hospital, Tampere, Finland

^c^Faculty of Medicine and Health Technology, Tampere University, Tampere, Finland

^d^Department of Internal Medicine/Respiratory Medicine and Allergology, The Sahlgrenska Academy, University of Gothenburg, Gothenburg, Sweden

^e^Department of Paediatrics, Sahlgrenska Academy, University of Gothenburg, Gothenburg, Sweden

^f^Department of Public Health and Clinical Medicine, Umeå University, Umeå, Sweden

^g^Department of Respiratory Medicine, Seinäjoki Central Hospital, Seinäjoki, Finland

Supplementary data:

**Methods**
**Assessment of sensitization and clinical allergy**

Sensitization was assessed through the determination of sIgE levels and/or skin prick tests for 11 aeroallergens. In summary, blood samples were procured during clinical visits and subsequently preserved at -80°C. An evaluation of IgE levels against a composite of aeroallergens (Phadiatop) was then undertaken. Individuals exhibiting titers of ≥0.35 kUA/L underwent supplementary measurements for IgE antibody levels against specific allergens within the composite mixture, including cat, dog, horse, house dust mite (*Dermatophagoides pteronyssinus*, *Dermatophagoides farinae*), mold (*Cladosporium herbarum*), birch, timothy grass, and mugwort. Quantification of IgE levels was executed using the ImmunoCAP™ system (Phadia AB, Uppsala, Sweden), where IgE values equal to or surpassing 0.35 kUA/L for an individual allergen were regarded as positive. The SPTs comprised a standard panel of 11 aeroallergens (ALK, Hørsholm, Denmark), administered after a minimum antihistamine withdrawal period of 72 hours. A positive result was defined as a mean wheal diameter ≥3 mm after 15 min. *Clinical allergy* was defined by the presence of allergic sensitization (positive SPT or sIgE to any allergen; atopy), and self-reported allergic symptoms attributable to the same allergen family. These symptoms, evaluated during the clinical interview prior to the sensitization test results, included ocular manifestations, nasal discomfort, various forms of allergic nasal expressions, pruritus in the oral or pharyngeal region, respiratory challenges, exacerbation of asthma symptoms, pruritic skin rash, and disruptions in gastrointestinal function.

**Measurement of lung function**

Spirometry measurements were conducted using a MasterScope spirometer (Jaeger, Höchberg, Germany), adhering to the guidelines stipulated by the ERS/ATS ^1,2^. The Global Lung function Initiative's normal equation for Caucasians was utilized for reference values ^3^, enabling the calculation of Forced Expiratory Volume in 1 second (FEV_1_) percentage of predicted normal value (FEV_1_% predicted). Participants were advised not to exercise strenuously or use tobacco products—including cigarettes, hookah, e-cigarettes, or snus—for at least one hour before the examination. Their medications are reviewed in advance and paused before the visit. Antihistamines and montelukast were stopped three days prior. Long-acting beta-agonists, long-acting muscarinic antagonists, and their combinations were stopped no less than 24 hours before the visit. In contrast, an 8-hour gap was considered sufficient for short-acting beta-agonists. However, participants were encouraged to continue using their inhaled corticosteroids if prescribed.

Reversibility testing was performed using 4 doses of salbutamol (4x 100 ug = 400 ug) given 15 minutes after the pre-bronchodilator spirometry. A positive reversibility test is defined as an increase in FEV1 of ≥12% and ≥ 200 ml after bronchodilator use. Those who underwent methacholine challenge test (MCT) were excluded from calculation of post-bronchodilator values.

Methacholine challenge (MCT) was executed using Omron Ultrasonic Nebulizer NE-U07 (OMRON Corporation, Kyoto, Japan) adhering to ERS guidelines ^4^. Methacholine challenge testing was performed using sodium chloride (NaCl) solution as a negative control, and methacholine chlorine at cumulative doses 35.25, 105.75, 176.25, 528.75, 1233.75 and 1938.75 nmol. Spirometry was performed before and after each dose. If a 20% decrease in FEV_1_ was observed after the methacholine doses (compared to the post-saline measurement), the test was stopped. Four doses of salbutamol (4 x 100 ug = 400 μg) and four doses of ipratropium bromide (4 x 20 ug = 80 μg) was then given. If the participant’s FEV_1_ had not recovered to at least 90% of the baseline value after 30 minutes, two additional doses of salbutamol were administered. The bronchodilatation response was recorded but was not used for analysis in this study. The methacholine dose that causes a 20% drop in FEV_1_ is called PD20. The bronchial challenge was considered negative if there was not a 20 percent fall in FEV_1_ at the cumulated dose of 1,938.75 nmol of methacholine. A PD20 ≤ 1,938.75 nmol of methacholine chloride indicates borderline, a PD20 130-500 nmol as mild, 30-130 nmol as moderate and a PD20 <30 nmol of methacholine was considered as severe hyperreactivity ^4^

**Definitions of diseases**

*Asthma* was defined as meeting any of the following criteria: 1) documented diagnosis of asthma by a healthcare professional or a history of asthma accompanied by respiratory symptoms or use of asthma medication in the past 12 months; 2) respiratory symptoms or use of asthma medication in the past 12 months, coupled with a positive reversibility test (12% and 200 ml increase in FEV_1_); or 3) respiratory symptoms or use of asthma medication in the past 12 months, accompanied by a positive methacholine challenge indicative of asthma. *COPD* was defined by a post-bronchodilator FEV_1_/Forced Vital Capacity (FEV_1_/FVC) ratio of less than 0.7 ^5^, along with a smoking history of 10 or more pack-years. *Metabolic disease* was defined as having obesity (body mass index [BMI] ≥30 kg/m2) or any of the following self-reported conditions: hypertension, hyperlipidaemia, or diabetes ^6^.

**Results:**


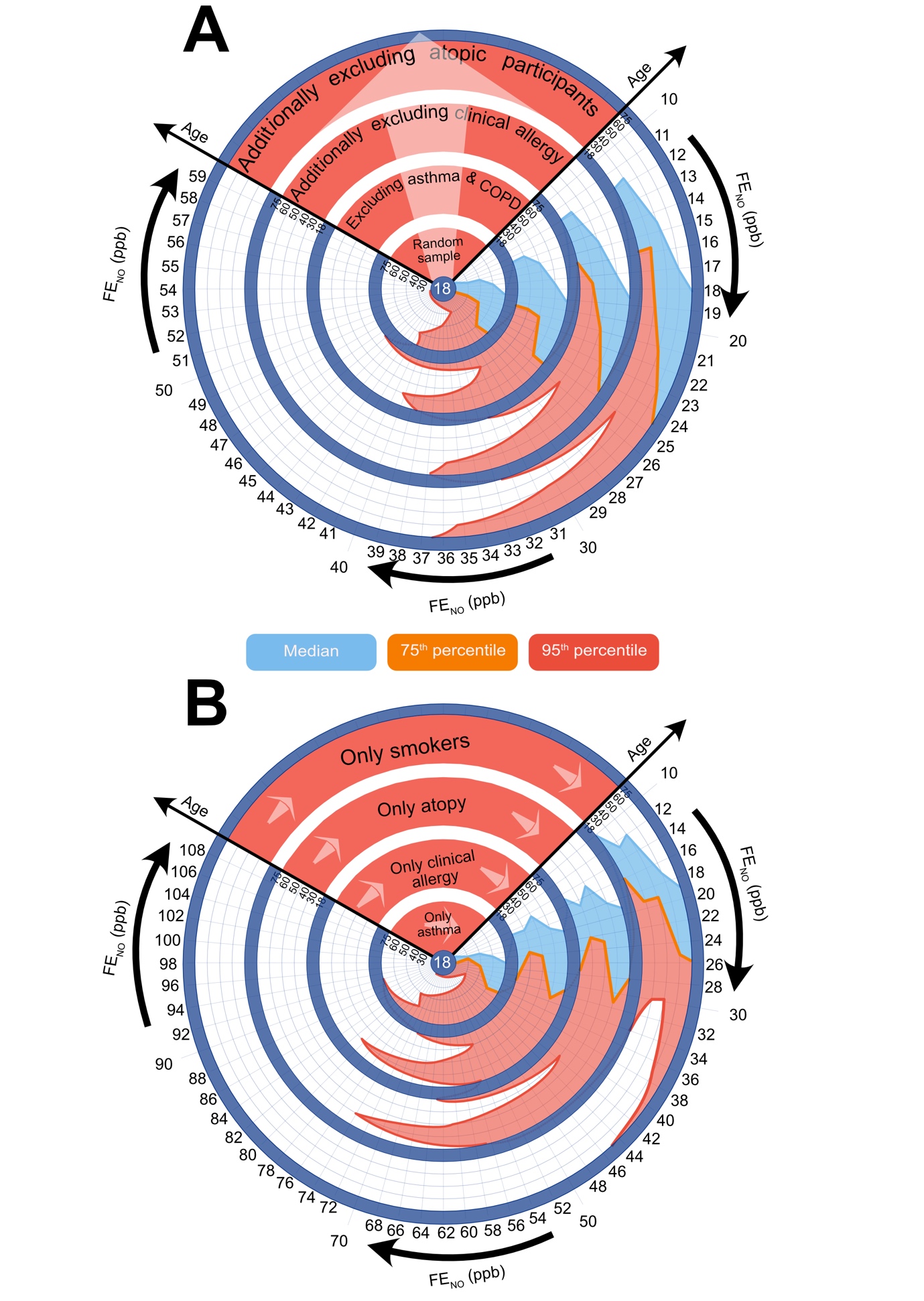


**Figure S1:** illustrates the characteristics of fractional exhaled nitric oxide (FE_NO_) across different age groups, delineated for: (A) the entire random sample (*N*=943), random sample participants excluding asthma and COPD patients (*n*=773), individuals additionally devoid of clinical allergy (*n*=587) , and individuals additionally devoid of atopy (*n=*519). (B) individuals with asthma (*n*=153), individuals with clinical allergy (*n*=276), atopic participants (*n*=349), and all smokers only (*n*=456). The depicted blue region demarcates the space between the median and 75^th^ percentile curves, while the red region signifies the interval between the 75^th^ percentile and 95^th^ percentile curves.

**Table S1: Factors associated with increased FE_NO_ levels in multiple linear regression analysis in participants devoid of clinical allergy, asthma and COPD.**

|  | Coefficient B | Std. Error | Lower 95% CI | Upper 95% CI | *p-* value |
| --- | --- | --- | --- | --- | --- |
| **Demographics** |  |  |  |  |  |
| Age | 0.459 | 0.017 | 0.426 | 0.492 | **<0.001** |
| Gender | -1.745 | 0.155 | -2.049 | -1.441 | **<0.001** |
| Height | 0.330 | 0.024 | 0.284 | 0.377 | **<0.001** |
| Weight | -0.295 | 0.022 | -0.338 | -0.251 | **<0.001** |
| BMI | 0.945 | 0.066 | 0.816 | 1.074 | **<0.001** |
| Ever smoking | -0.045 | 0.068 | -0.179 | 0.089 | 0.511 |
| Current smoking | -3.543 | 0.110 | -3.759 | -3.328 | **<0.001** |
| **Comorbidities** |  |  |  |  |  |
| Hypertension | 1.094 | 0.081 | 0.936 | 1.253 | **<0.001** |
| Diabetes | -3.979 | 0.205 | -4.382 | -3.577 | **<0.001** |
| **Lung function*** |  |  |  |  |  |
| Pre-bronchodilator FEV_1_ pred % | -0.647 | 0.081 | -0.805 | -0.489 | **<0.001** |
| Pre-bronchodilator FVC | -3.089 | 1.096 | -5.238 | -0.941 | **0.005** |
| Pre-bronchodilator FVC pred % | 0.845 | 0.069 | 0.710 | 0.981 | **<0.001** |
| Pre-bronchodilator FEV_1_/FVC | 77.499 | 7.806 | 62.198 | 92.799 | **<0.001** |
| Post-bronchodilator FEV_1_ | -4.118 | 0.631 | -5.355 | -2.880 | **<0.001** |
| Post-bronchodilator FEV_1_ pred % | 0.556 | 0.086 | 0.387 | 0.725 | **<0.001** |
| Post-bronchodilator FVC | 9.770 | 1.057 | 7.698 | 11.841 | **<0.001** |
| Post-bronchodilator FVC pred % | -0.854 | 0.065 | -0.982 | -0.726 | **<0.001** |
| Post-bronchodilator FEV_1_/FVC | -38.490 | 7.748 | -53.677 | -23.303 | **<0.001** |
| Post-bronchodilator -pre-bronchodilator FEV_1_ | -14.829 | 1.509 | -17.786 | -11.872 | **<0.001** |
| Reversibility % | 0.364 | 0.051 | 0.264 | 0.463 | **<0.001** |
|  |  |  |  |  |  |
| **R**: 0.447, R Square: 0.200, Adjusted R Square: 0.200 | | | | | |

Variables linked with the elevation of FE_NO_ were examined through linear regression analyses encompassing a cohort devoted from asthma, COPD and allergies (n = 519). FEV1: Forced expiratory volume in 1 second, FEV1%: Percentage of predicted normal value, FVC: Forced vital capacity. *Pre-bronchodilator FEV_1_, pre-bronchodilator FEV_1_/FVC, and Post-bronchodilator FEV_1_/FVC predicted % variables were excluded from the regression analysis due to multicollinearity reasons. Data are presented as the unstandardized b coefficient, standard error of b, and 95% CI.

References:

1. Graham BL, Steenbruggen I, Miller MR, Barjaktarevic IZ, Cooper BG, Hall GL, et al. Standardization of Spirometry 2019 Update. An Official American Thoracic Society and European Respiratory Society Technical Statement. Am J Respir Crit Care Med. 2019;200:e70–88.

2. Stanojevic S, Kaminsky DA, Miller MR, Thompson B, Aliverti A, Barjaktarevic I, et al. ERS/ATS technical standard on interpretive strategies for routine lung function tests. Eur Respir J. 2022;60:2101499.

3. Quanjer PH, Stanojevic S, Cole TJ, Baur X, Hall GL, Culver BH, et al. Multi-ethnic reference values for spirometry for the 3-95-yr age range: the global lung function 2012 equations. Eur Respir J. 2012;40:1324–43.

4. Coates AL, Wanger J, Cockcroft DW, Culver BH, Force the BTT, Carlsen KH, et al. ERS technical standard on bronchial challenge testing: general considerations and performance of methacholine challenge tests. European Respiratory Journal [Internet]. 2017 [cited 2024 Aug 19];49. Available from: https://erj.ersjournals.com/content/49/5/1601526

5. GOLD GI for COLD. Global Strategy for the Diagnosis, Management, and Prevention of Chronic Obstructive Pulmonary Disease [Internet]. 2023. Report No.: 2023. Available from: https://goldcopd.org/2023-gold-report-2/

6. Zakir F, Mohapatra S, Farooq U, Mirza MohdA, Iqbal Z. Chapter 1 - Introduction to metabolic disorders. In: Dureja H, Murthy SN, Wich PR, Dua K, editors. Drug Delivery Systems for Metabolic Disorders [Internet]. Academic Press; 2022 [cited 2024 Jul 17]. p. 1–20. Available from: https://www.sciencedirect.com/science/article/pii/B9780323996167000013
